# Verification of Argentine ant defensive compounds and their behavioral effects on heterospecific competitors and conspecific nestmates

**DOI:** 10.1038/s41598-018-19435-6

**Published:** 2018-01-24

**Authors:** Kevin F. Welzel, Shao Hung Lee, Aaron T. Dossey, Kamlesh R. Chauhan, Dong-Hwan Choe

**Affiliations:** 1 Department of Entomology, University of California, Riverside, CA 92521 USA; 2 Department of Entomology, University of California, Riverside, CA 92521 USA; 3 All Things Bugs LLC. 2211 Windsong Dr., Midwest City, OK 73130 USA; 4 Invasive Insects Biocontrol and Behavior Laboratory, USDA-ARS, BARC-West Bldg. 007, 10300 Baltimore Avenue, Beltsville, MD 20705 USA; 5 Department of Entomology, University of California, Riverside, CA 92521 USA

## Abstract

The invasive Argentine ant (*Linepithema humile*) has become established worldwide in regions with Mediterranean or subtropical climates. The species typically disrupts the balance of natural ecosystems by competitively displacing some native ant species via strong exploitation and interference competition. Here we report that Argentine ants utilize glandular secretions for inter and intra-specific communications during aggressive interactions with a heterospecific competitor, California harvester ant (*Pogonomyrmex californicus*). Chemical analyses indicated that Argentine ants deploy glandular secretions containing two major volatile iridoids, dolichodial and iridomyrmecin, on the competitor’s cuticular surface during aggressive interactions. Bioassays indicated that the glandular secretions function as a defensive allomone, causing high levels of irritation in the heterospecific. Furthermore, the same glandular secretions elicited alarm and attraction of conspecific nestmates, potentially enabling more rapid/coordinated defense by the Argentine ants. Two major volatile constituents of the glandular secretion, dolichodial and iridomyrmecin, were sufficient to elicit these responses in conspecifics (as a mixture or individual compounds). The current study suggests that invasive Argentine ants’ superior exploitation and interference competition may rely on the species’ effective semiochemical parsimony.

## Introduction

From its native range in South America, the Argentine ant, *Linepithema humile* (Mayr) (Hymenoptera: Formicidae) has become a global invader, particularly in areas with Mediterranean climates^1^. Argentine ants have the ability to interact and freely mix over long distances, form super colonies, and maintain a high rate of reproduction via multiple queens^2^. These traits of the invasive Argentine ant, unicoloniality and polygyny, have likely contributed to their successful establishment in locations around the globe^3,4^. The successful establishment of Argentine ant populations in their non-native ranges typically results in numerous detrimental effects, such as reduction of arthropod diversity^5^, proliferation of phloem-feeding plant pests^6,7^, competition with pollinators^8,9^, and invasion of residential structures^10^.

Once introduced into new habitats, Argentine ants must compete with existing native species to successfully establish^11^. Exploitation competition (i.e., depleting resources that would otherwise be used by other species) and interference competition (i.e., prohibiting access to resources by other species via direct interactions) have been studied to understand their roles in the Argentine ant’s successful establishment in non-native habitats, especially against other native ant species^2,12,13^. Some of these empirical studies have indicated that invasive populations of Argentine ants have a higher competitive ability than most native ant species in exploitation and interference competition, not only discovering and dominating resources more quickly than the native ants, but also outcompeting native ants via highly aggressive interactions at the resources^2,14^.

In many social insects, semiochemicals play an integral role in organizing colony-level activities such as foraging and defense^3,15^. Argentine ants’ high competitiveness in exploitation and interference competition might be, at least in part, due to their efficiency in communicating via semiochemicals^16^. For example, the rapid mass-recruitment of nestmates to the new resource via the use of trail pheromone may have contributed to their success in exploitation competition by allowing them to numerically dominate the resource more quickly than competitors^2,17^. Dolichodial, iridomyrmecin, and (Z)-9-hexadecenal have been studied for their potential function in trail-following and recruitment behavior of Argentine ants^18,19^. Argentine ants also defend the resource from other competitors by directly preventing the competitors’ physical establishment at the resource^2^. For example, aggressive interactions between Argentine ants and other native ant species have been described in several studies^2,20–22^, suggesting that Argentine ants’ high competitiveness in aggressive interactions might play a role in their ability to outcompete and displace native ant species.

**Figure 1:**
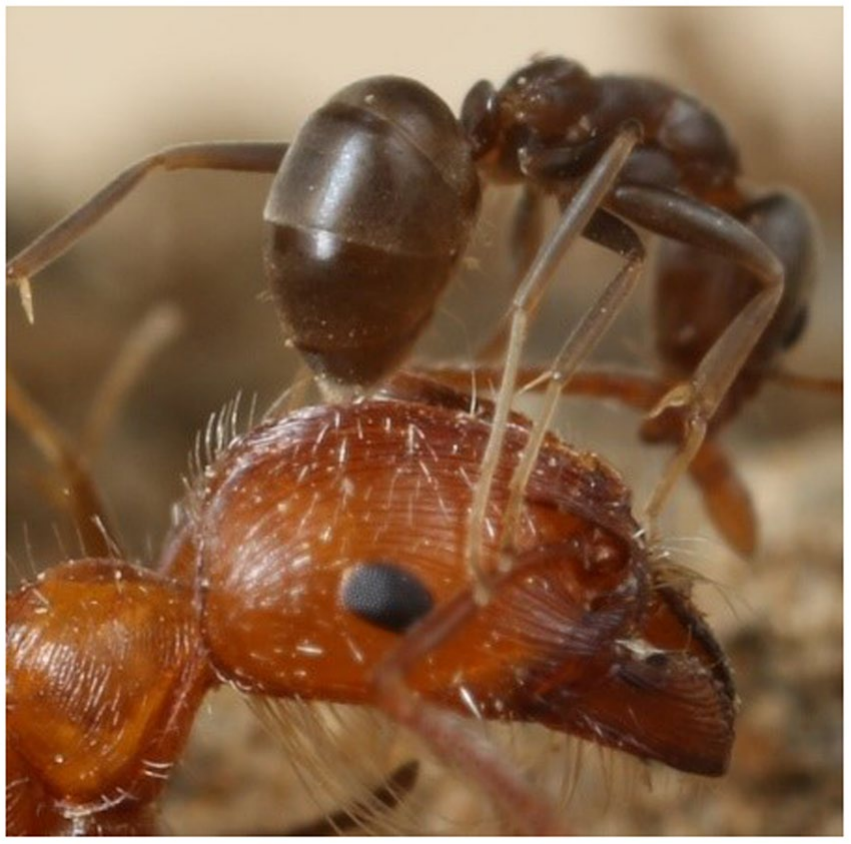
An Argentine ant, *Linepithema humile*, worker performing gaster bending behavior on the head of a harvester ant worker, *Pogonomyrmex californicus*. Photo by Dr. Dong-Hwan Choe.

Previous studies have implied that Argentine ants deploy defensive compounds during aggressive interactions with other species of ants^14,21,23,24^. In particular, Argentine ants have been observed to display a behavior known as “gaster bending” or “gaster flexing” during aggressive interactions^25,26^. In this behavior, a worker Argentine ant typically bends its gaster ventrally to place the tip of the gaster onto the opponent (Fig. 1). It was also speculated that irritant chemicals might be “sprayed” on the opponent via similar behaviors^27^. Several studies have suggested that Argentine ants might use pygidial (anal) gland compounds for defensive functions, like other dolichoderine ants^3,28,29^. Historically, the pygidial glands of dolichoderine ants have been believed to produce defensive secretions that provoke the alarm response, or chemicals with antibiotic/insecticidal effects^30–33^. Two iridoids, dolichodial and iridomyrmecin, have been described in the pygidial gland secretions of Argentine ant^28,30^. However, detailed behavioral and chemical investigations have not been conducted to elucidate the chemical ecology of Argentine ant aggression and the possible defensive functions of the pygidial gland chemicals.

Competition between Argentine ants and harvester ants (*Pogonomyrmex* spp.) has been documented in field sites in California, and typically resulted in the displacement or decline of harvester ant populations in areas with both species^34,35^. The competitive relationship between these two species is unique because they naturally occupy different ecological niches. Argentine ants prefer relatively high moisture content in the soil for nesting, and they are omnivorous, collecting insect prey and tending phloem-feeding insects for their sugary secretions^34^. In contrast, harvester ants tolerate higher temperatures and drier conditions in semi-desert habitats, primarily relying on diets consisting of gathered seeds^34,36^. However, Argentine ant populations often penetrate drier semi-desert areas via surrounding urban edges where they become abundant due to irrigation from the developed areas^37,38^. In addition, coastal environments with fresh water from natural springs or ephemeral waterways may provide suitable environmental conditions for Argentine ant establishment^39^. This habitat overlap between harvester ants and the Argentine ants results in competitive interactions between the two species^38,40^. Nevertheless, due to differences in their preferred diets and nesting conditions, competition for identical food resources and nest sites do not appear to be among the main reasons for the decline of harvester ant populations^34,41^. Instead, competitive interactions between these species often take the form of nest raiding by Argentine ants, which also has been observed between Argentine ants and other native ants^14,42^. In California, invasive Argentine ant populations have been observed to exploit the brood of *Pogonomyrmex subnitidus* (Emery) colonies, resulting in the eventual decline of harvester ant populations^41^.

The present study explored the chemical ecology of aggressive interactions between Argentine ant and the California harvester ant, *Pogonomyrmex californicus* (Buckley). In particular, we hypothesized that Argentine ants release and apply pygidial gland secretions through the gaster bending response during aggressive interactions with other species. By exploring the functions of the pygidial gland secretions (and chemicals) in Argentine ants’ competitive interactions with heterospecific competitors, we attempted to elucidate some of the key mechanisms behind their remarkable competitive ability. The objectives of this study were: (1) to determine if the pygidial gland secretion is produced by Argentine ants during aggressive interactions with harvester ants, (2) to determine if the pygidial gland secretion is applied on the harvester ants’ cuticle during aggressive interactions, (3) to understand the behavioral effects of the crude pygidial secretion on the harvester ant when it is topically applied to the cuticle, and (4) to understand the behavioral effects of the crude pygidial secretion and its volatile constituents on Argentine ants when the chemicals are presented from a nearby point source.

## Results

### Headspace volatile analyses

**Figure 2:**
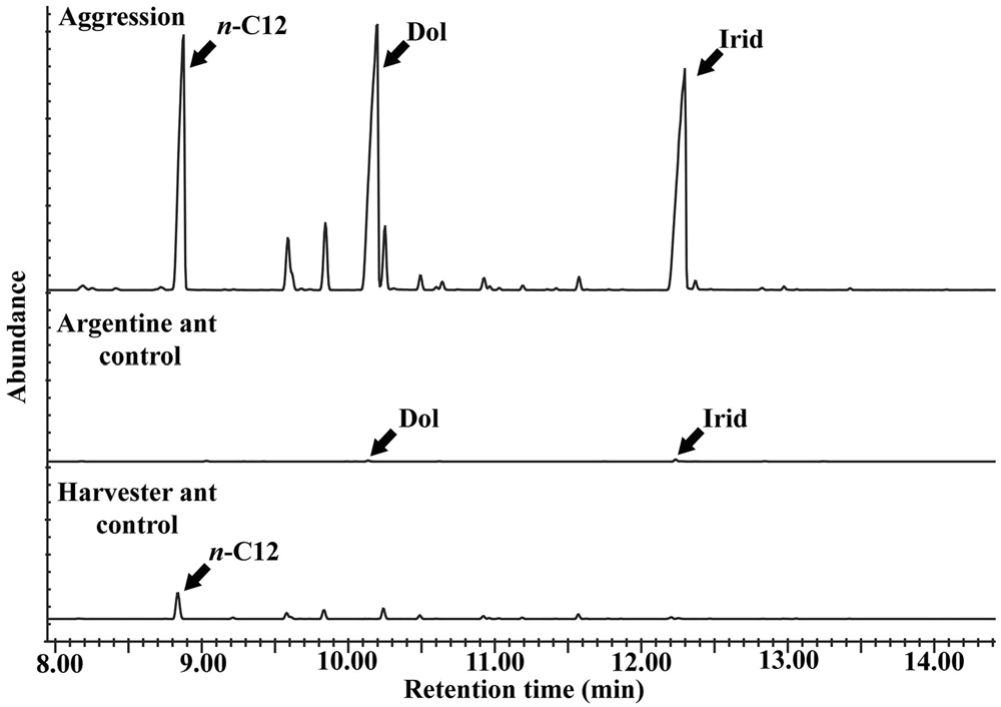
Overlay of representative chromatograms (GC-MS) from the headspace volatile analyses. The sample of volatiles were collected from vials containing five Argentine ants and one harvester ant (Aggression), five Argentine ants only (Argentine ant control), or one harvester ant (Harvester ant control). X-axis represents retention time (min) and y-axis represents relative abundance. Dolichodial (Dol) and Iridomyrmecin (Irid) were consistently detected in all Aggression samples (n = 10) and Argentine ant controls (n = 5). Harvester ant controls did not show the presence of dolichodial or iridomyrmecin (n = 5). *n*-Dodecane (*n*-C12) was present in Aggression and Harvester ant control samples. *n-*C12 was not present in the Argentine ant control samples.

Headspace volatile samples were analyzed from the following samples using solid phase microextraction (SPME) and gas chromatography – mass spectrometry (GC-MS): vials with Argentine ants and single harvester ant (Aggression), vials with Argentine ants only (Argentine ant control), and vials with single harvester ant only (Harvester ant control). Agonistic responses including Argentine ants’ gaster bending were observed in all vials containing both species of ants. The headspace volatile analyses indicated that two iridoids, dolichodial and iridomyrmecin, were consistently present in relatively large quantities in the vials where there were aggressive interactions between Argentine ants and harvester ants (Fig. 2, Aggression, 10 of 10). The iridoids were also detected in the headspaces of the vials containing Argentine ants only (Fig. 2, Argentine ant control, 5 of 5), but in significantly smaller quantities when compared with the former. The comparisons between integration values of the corresponding iridoids indicated that much higher amounts of iridoids (i.e., 193–197 times larger integration value) were produced by the Argentine ants during aggression (n = 10) compared to the Argentine ant control (n = 5) [dolichodial: 2.9 × 10^7^ ± 1.1 × 10^5^ vs. 1.5 × 10^5^ ± 8.7 × 10^4^ (mean ± SEM) for aggression and control, respectively; iridomyrmecin: 7.9 × 10^7^ ± 5.5 × 10^7^ vs. 4.0 × 10^5^ ± 1.0 × 10^5^ for aggression and control, respectively] (Wilcoxon Rank Sum Test: *z* = 2.76, *P* = 0.003 for dolichodial and *z* = 2.76, *P* = 0.006 for iridomyrmecin). The iridoids were not detected in the vials containing harvester ant only (Fig. 2, harvester ant control, n = 5). All negative control (i.e., empty) vials did not contain any detectable peaks (n = 5). A third peak, identified as *n*-dodecane, was present in relatively large quantities in both aggression and harvester ant control samples, but not in Argentine ant control samples (Fig. 2), indicating that it was likely produced by the harvester ants. However, further testing would be necessary to confirm this. The overall data indicated that the Argentine ants released significantly larger quantities of pygidial gland chemicals when they were actively engaged in aggressive interactions with the harvester ants.

### Cuticular extract analyses

**Figure 3:**
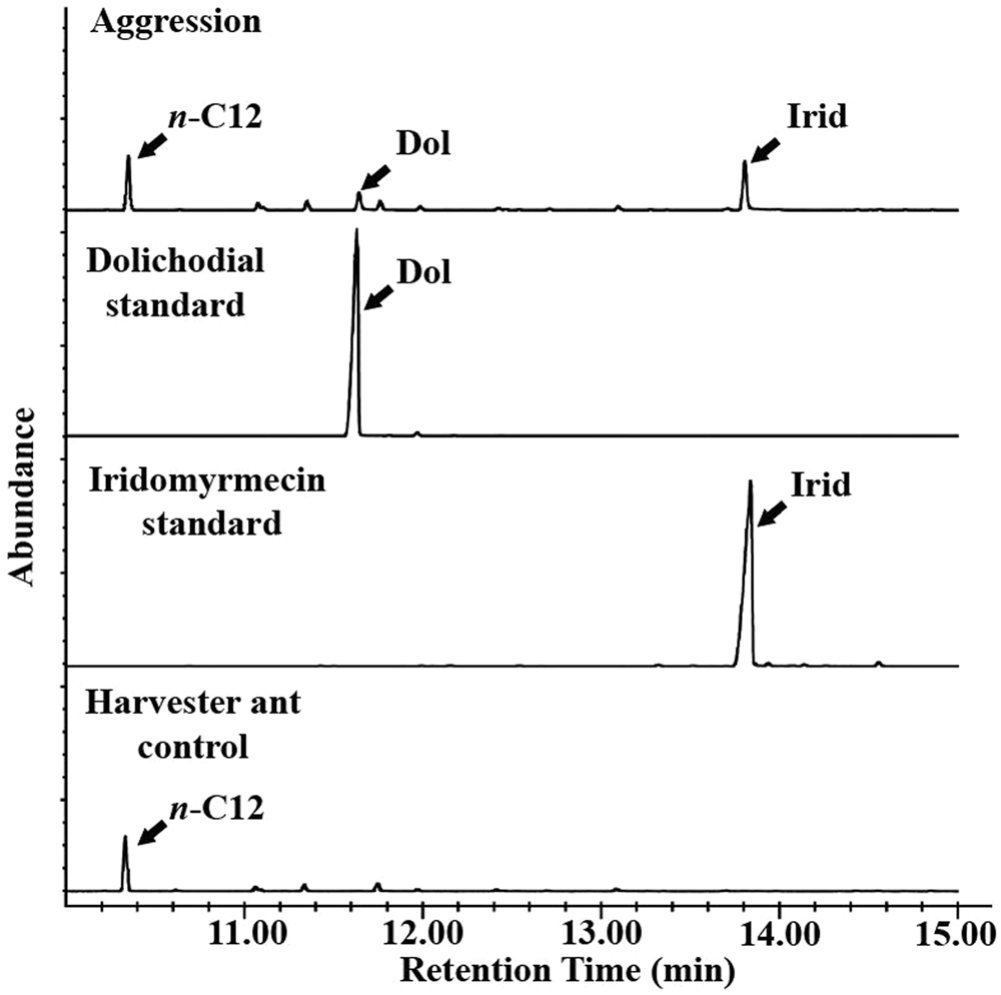
Overlay of representative chromatograms (GC-FID) from the cuticular extract analyses. The samples were collected from a pooled sample of 30 harvester ants after Argentine ant aggression (Aggression), two standards for identification (map of the first two principal componentscin standard), and the cuticular extract of harvester ants without Argentine ant aggression (Harvester ant control). X-axis represents retention time (min) and y-axis represents relative abundance. Peaks for dolichodial (Dol) and iridomyrmecin (Irid) are marked with arrows. *n-*Dodecane (*n*-C12) was only present in “Aggression” and “Harvester ant control” samples. Chromatograms represent the polar fractions (methylene chloride).

The gas chromatography-flame ionization detector (GC-FID) analyses of the cuticular extracts indicated that dolichodial and iridomyrmecin were consistently detected in the polar fraction (methylene chloride fraction) of the harvester ant cuticular extracts after the gaster bending of the Argentine ants (Fig. 3, Aggression). The nonpolar fraction (hexane fraction) mostly consisted of cuticular hydrocarbons, without detectible amounts of the iridoids. The cuticular extracts of the control harvester ants (without Argentine ants’ aggression) did not contain dolichodial and iridomyrmecin in either the polar or nonpolar fractions. However, *n*-dodecane was present in the control harvester ants’ cuticular extracts (Fig. 3, Harvester ant control). During the 2-min observation period, the total number of the gaster bending responses on a single harvester ant was 16.0 ± 1.1 (mean ± SEM, n = 30). The estimated amounts of dolichodial and iridomyrmecin on the cuticle of a single harvester ant were 0.08 and 0.28 µg, respectively (ratio 1:3.5), calculated from a pool of 30 harvester ants.

### Collection and chemical analyses of pygidial gland chemicals

Pygidial gland secretion was collected from Argentine ant foragers by gently squeezing the dorsal posterior region of the gaster with a pair of fine forceps. This crude secretion was referred to as “pygidial gland extract” or “PGE” hereafter. One ant equivalent of PGE weighed 17.6 ± 2.0 µg (mean ± SEM, n = 10) and contained ≈0.05 µg of dolichodial and ≈0.15 µg of iridomyrmecin (ratio 1:2.8). These values were comparable with the quantities of the iridoids estimated for one harvester ant cuticle, justifying the use of one ant equivalent of Argentine ant PGE in the subsequent bioassays. The two iridoids (≈0.2 µg total) accounted for approximately 1.2% of the total weight of PGE.

### Response of harvester ants when topically treated with pygidial gland chemicals

Behavioral responses of harvester ants topically treated with Argentine ant PGE (one ant equivalent) were distinctively different from those of the untreated control group. Overall, the amounts of time spent in different behavioral categories differed significantly between the treatment (n = 10) and control (n = 10) groups (Permutation MANOVA: *F* = 49.9, df = 1,19, *P* < 0.001). Harvester ant mortality after 24 h was 20% (2/10) and 10% (1/10) for treatment and control groups, respectively.

**Figure 4:**
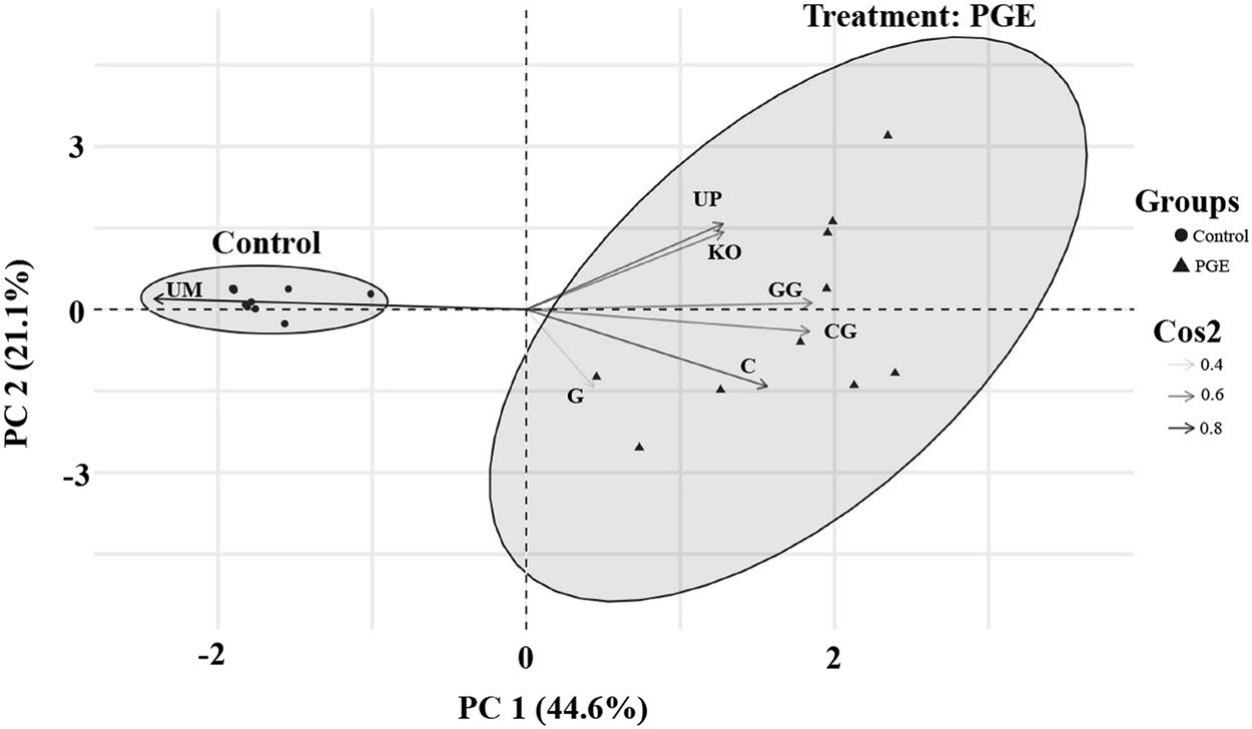
A factor map of the first two principal components PC1 and PC2. Individual harvester ant timed behavioral response categories include: uninhibited mobility (UM), mandible clasping (C), mandible clasping with gaster manipulation (CG), grooming (G), grooming with gaster manipulation (GG), turning upside down, (UP), and knockout (KO). Shaded ellipses illustrate groupings corresponding to PGE treatment (triangles) and control (circles) harvester ants. Arrows represent squared cosine (Cos2) values of behavioral response for a given observation. Behavior responses with Cos2 closer to 1 (darker arrows) are considered important components for that group.

The principal component analysis (PCA) of individual harvester ant timed behavioral responses showed a clear discrimination between the treatment and control groups (Fig. 4). Two principal components (PC) with eigenvalues greater than one [PC 1 (3.12) and PC 2 (1.48)] accounted for 66% of the total variation. PC 1 indicated that “uninhibited mobility” was negatively correlated with all other timed behavioral responses. Harvester ants in the control group spent a total of 95% of their 10 min trial period performing “uninhibited mobility” while harvester ants in the treatment group only spend 6% of their time in this behavioral response category (Fig. 4, see Cos2 values). Instead, harvester ants in the treatment group primarily performed behaviors that represented irritation and insecticidal effects, which accounted for 94% of the total 10 min trial period. For example, the average durations for “upside-down” and “knockout” responses of harvester ants in the treatment group were 153.7 ± 75.9 s and 186.9 ± 76.3 s (mean ± SEM, n = 10), respectively. Harvester ants in the control group did not show “upside-down” and “knockout” responses.

### Response of Argentine ants to the items treated with pygidial gland chemicals

**Figure 5:**
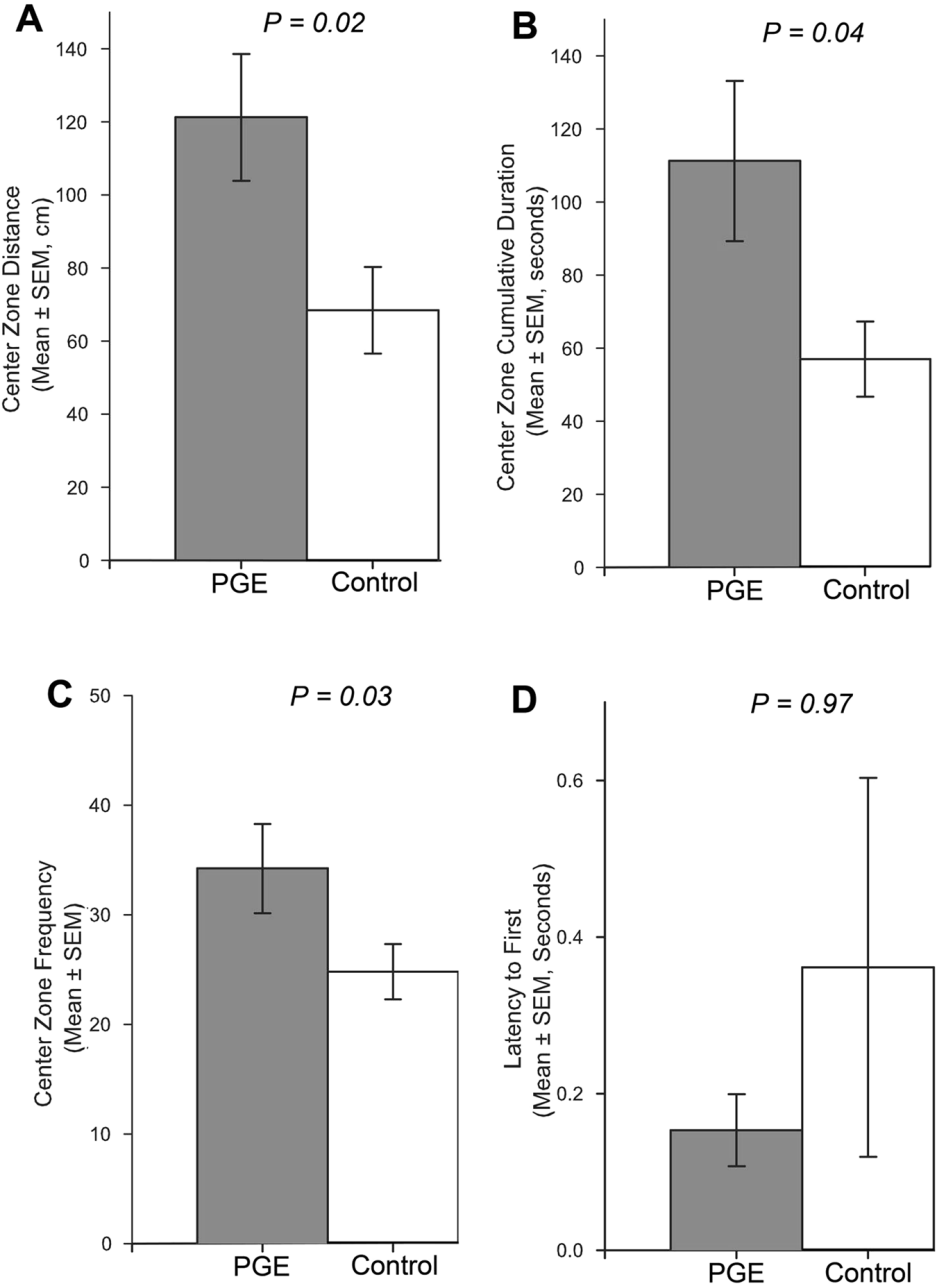
Responses of an Argentine ant to a fixed dead harvester ant with or without Argentine ant pygidial gland extract (PGE). Ten replications were made for PGE treatment (grey) and control (white). The behavioral parameters analyzed include: center zone distance (**A**), center zone cumulative duration (**B**), center zone frequency (**C**), and latency to first (**D**). P-value is provided for each behavioral parameter (see results for the statistical methods used). Error bars indicate SEM values.

The behavioral response parameters of the Argentine ant to a dead harvester ant fixed in the arena center are summarized in Fig. 5 and Supplementary Fig. 2. Argentine ants in the PGE treatment and control were similar in their overall speed [1.65 ± 0.12 and 1.48 ± 0.11 cm s^−1^ (mean ± SEM, n = 10) for PGE and control, respectively] (two-sided two-sample t-test: *T* = −1.01, df = 18, *P* = 0.33) (Supplementary Fig. 2A), and overall travel distance (980.7 ± 71.2 and 891.3 ± 66.2 cm for PGE and control, respectively) (two-sided two-sample t-test: *T* = −0.92; df = 18; *P* = 0.37) (Supplementary Fig. 2B).

However, Argentine ants travelled significantly longer distances in the center zone when the center zone had the PGE-treated harvester ant compared to the untreated control harvester ant (center zone distance: 121.2 ± 11.5 and 76.1 ± 14.8 cm for PGE and control, respectively) (two-sided two-sample t-test: *T* = −2.4, df = 18, *P* = 0.02) (Fig. 5A). Center zone velocities of the ants were not significantly different between PGE treatment and control (center zone velocity: 1.38 ± 0.11 and 1.37 ± 0.14 cm s^−1^ for PGE and control, respectively) (two-sided two-sample t-test: *T* = −0.03, df = 18, *P* = 0.99) (Supplementary Fig. 2C). When the center zone had PGE-treated harvester ants, the Argentine ants spent significantly longer time in the center zone compared to control (center zone cumulative duration: 114.5 ± 20.0 s and 58.5 ± 9.3 s for PGE and control, respectively) (two-sided Welch two-sample t-test: *T* = −2.5, df = 18, *P* = 0.04) (Fig. 5B). Total number of visits to center zone was also significantly greater in the assays with PGE-treated harvester ants compared to controls (center zone frequency: 34.2 ± 4.1 and 24.8 ± 2.5 for PGE and control, respectively) (two-sided two-sample t-test: *T* = 2.0, df = 18, *P* = 0.03) (Fig. 5C). Times until the first visit to the center zone were not significantly different between PGE treatment and control (latency to first: 0.15 ± 0.05 and 0.36 ± 0.24 s for PGE and control, respectively) (Wilcoxon rank sum test: *z* = 0, df = 18, *P* = 0.97) (Fig. 5D).

**Figure 6:**
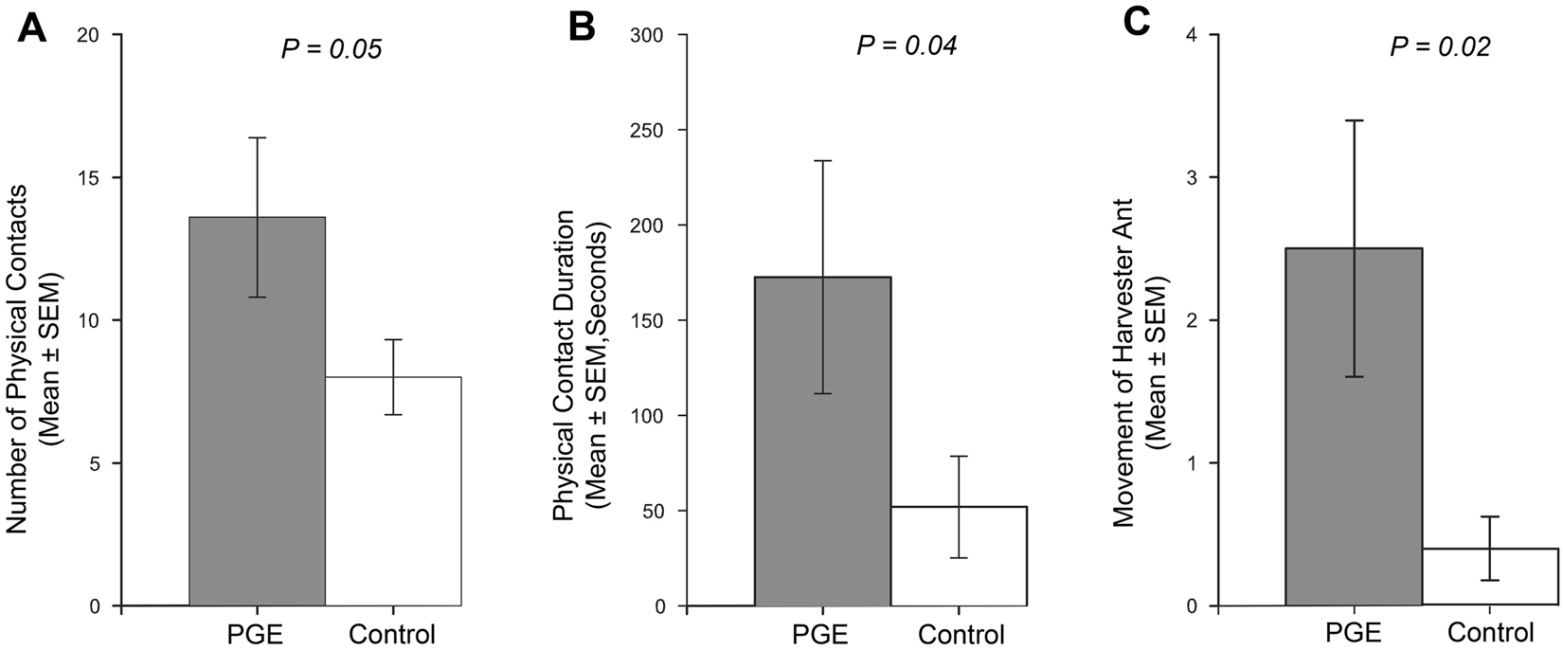
Response of an Argentine ant to an unfixed dead harvester ant with or without Argentine ant pygidial gland extract (PGE). Ten replications were made for PGE treatment (grey) and solvent only control (white). The Behavioral parameters analyzed include: number of physical contacts (**A**), physical contact duration (**B**), and movement of harvester ant (**C**). P-value is provided for each behavioral parameter (see results for the statistical methods used). Error bars indicate SEM values.

The behavioral response parameters of Argentine ant to an unfixed dead harvester ant are summarized in Fig. 6 and Supplementary Fig. 3. Argentine ants in PGE treatment and control were similar in their overall speed [1.17 ± 0.22 and 1.56 ± 0.15 cm s^−1^ (mean ± SEM, n = 10) for PGE and control, respectively] (two-sided two-sample t-test: *T* = 1.5, df = 18, *P* = 0.15) (Supplementary Fig. 3A), and overall travel distance (701.6 ± 129.5 and 937.4 ± 89.2 cm for PGE and control, respectively) (two-sided two-sample t-test: *T* = 1.5, df = 18, *P* = 0.15) (Supplementary Fig. 3B).

However, number of physical contacts between Argentine ant and the dead harvester ant was significantly greater in the PGE treatment compared to control (13.6 ± 2.8 and 8.0 ± 1.3 for PGE and control, respectively) (two-sided Welch two-sample t-test: *T* = −1.8, df = 18, *P* = 0.05) (Fig. 6A). During the physical contact, Argentine ant showed antennation, mandible clasping, gaster bending, or crawling responses. Total duration of physical contact between Argentine ants and the dead harvester ant was significantly longer in the PGE-treated harvester ants compared to the control harvester ants (172.6 ± 61.2 and 51.9 ± 26.7 s for PGE and control, respectively) (Wilcoxon rank sum test: *z* = −2.1, df = 18, *P* = 0.04) (Fig. 6B). The Argentine ants also moved the PGE-treated dead harvester ants more frequently compared to the control harvester ants (2.5 ± 0.9 and 0.4 ± 0.2 for PGE and control, respectively) (Wilcoxon rank sum test: *z* = −2.3, df = 18, *P* = 0.02) (Fig. 6C).

**Figure 7:**
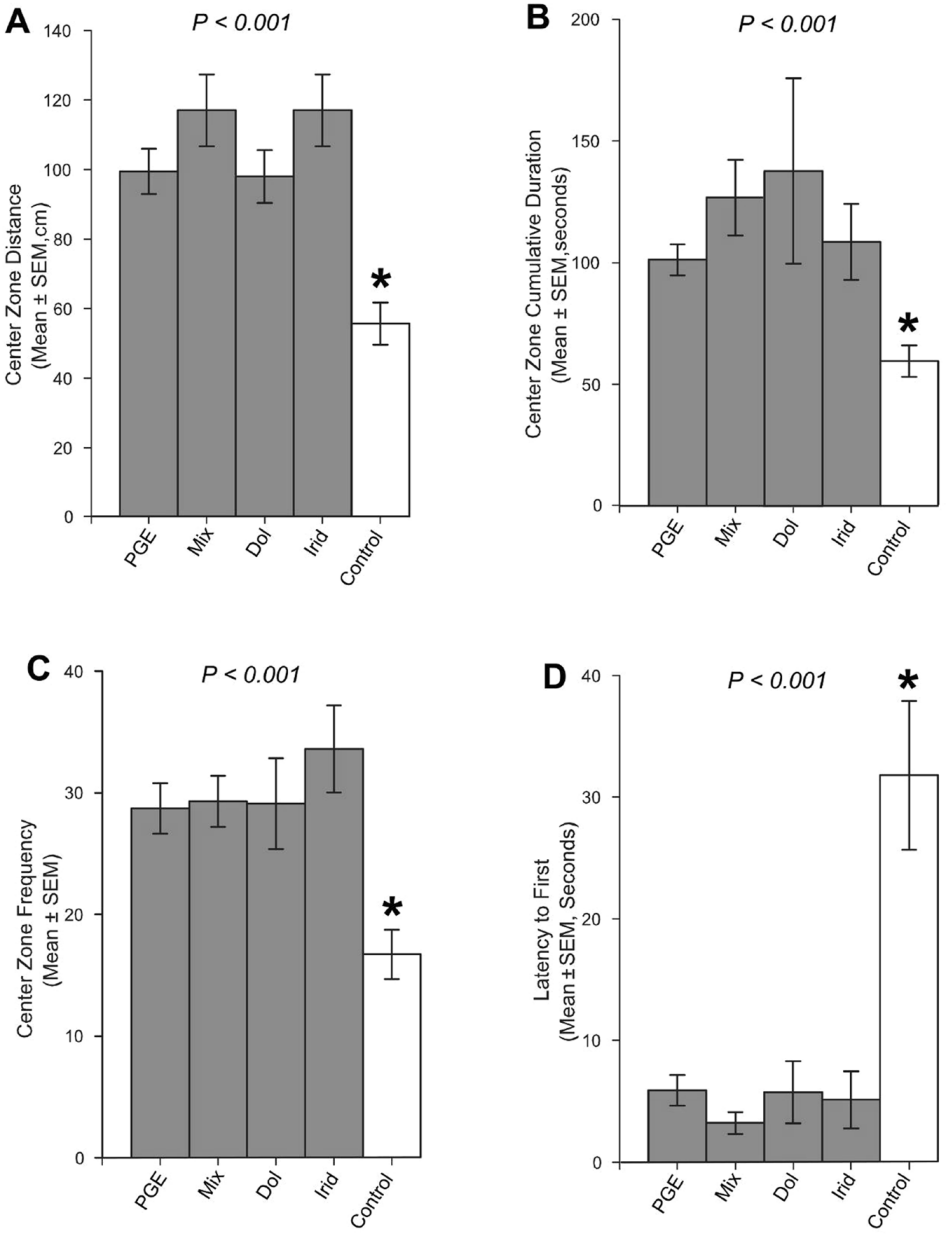
Response of an Argentine ant to a glass bead treated with pygidial gland extract (PGE), a mixture of authentic standards of dolichodial and iridomyrmecin (Mix), dolichodial standard (Dol), iridomyrmecin standard (Irid), and solvent only (Control). Ten replications were made for each of the treatments (grey) and control (white). The behavioral parameters analyzed include: center zone distance (**A**), center zone cumulative duration (**B**), center zone frequency (**C**), and latency to first (**D**). Asterisk (*) indicates control that was significantly different from all of the treatments. For multiple comparisons, Dunn’s test for all-pairwise comparisons of mean ranks with a Bonferroni adjustment was used (see results). P-value is provided for each behavioral parameter. Error bars indicate SEM values.

The behavioral response parameters of Argentine ant to a glass bead fixed in the arena center are summarized in Fig. 7, Supplementary Fig. 4, and Supplementary Table 1. Argentine ants in all treatments (n = 10 for each) and control (n = 10) were similar in their overall speed (Kruskal-Wallis test: *H* = 7.5, df = 4, *P* = 0.10) (Supplementary Fig. 4A), and overall travel distance (Kruskal-Wallis test: *H* = 7.6, df = 4, *P* = 0.10) (Supplementary Fig. 4B).

However, several behavioral parameters were significantly different between the treatments and control. When glass beads were treated either with PGE or with authentic standards of the iridoids (individually or as a mixture), Argentine ants traveled longer distances in the center zone compared to control (Dunn’s test: *Z* = 2.8, df = 4, *P* < 0.001) (Fig. 7A). However, their center zone velocities were similar across the treatments and control (Kruskal-Wallis test: *H* = 3.8, df = 4, *P* = 0.44) (Supplementary Fig. 4C). Furthermore, Argentine ants in the treatments (PGE or authentic standard of the iridoids) spent significantly longer time in the center zone (center zone cumulative duration) (Dunn’s test: *Z* = 2.8, df = 4, *P* < 0.001) (Fig. 7B), and entered the center zone more frequently (Dunn’s test: *Z* = 2.8, df = 4, *P* < 0.001) compared to control (Fig. 7C). Latency to the first entry into the center zone was also significantly shorter in the treatments compared to control (Dunn’s test: *Z* = 2.8, df = 4, *P* < 0.001) (Fig. 7D).

## Discussion

The successful establishment of Argentine ants in numerous locations worldwide is due, at least in part, to its superior ability to exploit and defend resources^2^. Their trail pheromones play a critical role in resource exploitation by efficiently leading colony members to the resources^43^. Once they arrive at the resources, they maintain their dominance against competing species through aggressive physical contact^21,42^. The current study confirms that Argentine ants utilize their pygidial gland secretions during aggressive physical contact with a heterospecific competitor. The pygidial gland secretions cause irritation and disorientation to competitors when topically applied on their cuticles. The pygidial gland secretion or its major volatile constituents, dolichodial and iridomyrmecin, also release alarm and aggregation responses for other Argentine ants nearby, potentially facilitating group defense.

Aggressive physical contact between Argentine ants and harvester ants resulted in the release of dolichodial and iridomyrmecin in the headspace. The same iridoids were also detected from the headspace of control vials containing Argentine ants only. However, the higher quantities of dolichodial and iridomyrmecin in the headspace were only associated with aggressive interactions. Our data suggest that Argentine ants release small amounts of dolichodial and iridomyrmecin even in the absence of aggression, but strongly increase their release during aggressive interactions. There are possible other behavioral functions for the iridoids at low concentrations. For example, the presence of dolichodial and iridomyrmecin on the cuticle of live Argentine ants inhibits necrophoric behaviors^44^. In addition, dolichodial and iridomyrmecin are among the chemical constituents of Argentine ant recruitment trails^19^.

Based on observations of Argentine ant gaster bending behavior and the response of competitor ants to this behavior, previous studies have inferred that Argentine ants deploy defensive compounds during aggressive interactions^2,14,21,23^. The current study experimentally confirmed that Argentine ants deploy dolichodial and iridomyrmecin from their pygidial glands and deposit them on the cuticles of opponents during aggression, through the gaster bending response. During the 2-min interaction between one harvester ant and a group of Argentine ant foragers (100), multiple gaster bending responses (≈16) were observed. Multiple applications of the pygidial chemicals might be necessary to overcome larger heterospecific competitors. In the current study, the harvester ant did not appear to be significantly affected by the first gaster bending response. However, after several additional gaster bending responses (towards the end of the 2-min observations), some evident behavioral effects (e.g., disorientation, signs of irritation) were observed in the harvester ants. One-on-one physical contact between Argentine ants and native species usually result in the death of the Argentine ant^2,42^, suggesting the importance of numerical dominance by the Argentine ant during aggressive physical contact involving the gaster bending response.

In addition to dolichodial and iridomyrmecin, *n*-dodecane was detected in some of the samples with the harvester ants. *n*-Dodecane was detected only in headspace volatiles from harvester ants, with larger quantities generally associated with aggression (Fig. 2). *n*-Dodecane was also detected in cuticular extracts of harvester ants (aggression as well as harvester ant controls) (Fig. 3). Thus, it is likely that *n*-dodecane originated from the harvester ants. *n*-Dodecane is the most abundant volatile component found in the Dufour’s gland of *Pogonomyrmex rugosus* and *P*. *barbatus*^45^. It has been suggested that a pheromone blend containing *n*-dodecane might be used for orientation on the homing course by harvester ants^46^. The production of *n*-dodecane and its potential functions in interspecific interactions of harvester ants (e.g., defense, alarm, etc.) warrant further investigations.

The topical application study confirmed the allomonic role of the Argentine ant pygidial gland secretions. In particular, the pygidial gland chemicals caused significant levels of irritation and disorientation in harvester ants when topically applied. The treated harvester ants typically showed continuous grooming and mandible clasping responses. In some cases, the treated harvester ant lost its ability to right itself, often resulting in going upside down or being temporarily incapacitated. Even if the current study did not examine the effects of pure iridoids on the harvester ants, it is likely that dolichodial and iridomyrmecin in the pygidial gland secretion are among the major causes for the behavioral responses observed in the treated harvester ants. Previous studies have reported that dolichodial and iridomyrmecin have “insecticidal” properties and might serve as defensive allomones in the Argentine ant and other species of ants^28–30^. The current study clearly shows that dolichodial and iridomyrmecin are the primary volatile chemical constituents of the pygidial gland secretion. However, further chemical investigation would be necessary to determine if there are other non-volatile and/or more polar compounds in the pygidial gland secretion, which may be bioactive. Besides the iridoids, which are responsible for ≈1.2% of the total weight of the secretion, the pygidial gland secretion also appears to contain a substantial amount of water (based on the result from tests of pygidial gland secretions with cobalt chloride treated paper; unpublished data).

It is also important to note that the “insecticidal” effects of the pygidial gland secretions (and associated iridoids) appear to be only temporary for the harvester ants. For example, eight out of the ten harvester ants recovered from the effects of topical application of the pygidial gland secretion after 10 min and subsequently showed uninhibited mobility. Some previous studies have stated that the toxicity of the iridoids are “not great”^29,47^; however, some of these studies did not provide detailed protocols for how toxicity was measured. Additional studies conducted with other biological systems utilizing identical or similar iridoid chemistries suggest that the iridoids might function as general “aggression suppressants” for ants in interspecific interactions. For example, a blend of iridomyrmecin and iridomyrmecin-like compounds were found in the mandibular gland secretions of the aphid hyperparasitoid *Alloxysta brevis*, and these secretions discourage aggression by aphid-tending ants (*Lasius* sp.)^48^. Iridodials and iridomyrmecin were found in the pygidial gland secretions of *Tapinoma* sp., which suppressed the aggression in heterospecific ants when they were daubed with the secretion^49^. The potential function of the pygidial gland chemicals as a general aggression suppressant in Argentine ants’ interspecific interactions warrants further research. Even though the current study did not continue tracking the competitive interactions between Argentine ants and the harvester ants after the treatment with the pygidial gland chemicals, it is possible that the irritation and insecticidal effects of the pygidial gland chemicals might provide an important advantage for the Argentine ants by temporarily disabling the harvester ants during competitive confrontations near resources.

Upon encountering harvester ants treated with the pygidial gland secretions (fixed or unfixed in the arena center), Argentine ants responded with several behavioral components that are typically associated with an alarm reaction. These behavioral components included attraction to the source of chemicals, increased distance traveled, and arrested motion while displaying aggressive responses such as mandible clasping and gaster bending^3,50,51,52^. The subsequent assays with inert glass beads treated with iridoid standards indicated that the volatile iridoids in the pygidial gland secretion are primarily responsible for Argentine ants’ alarm responses towards the treated items. Historically, the volatile compounds produced in the pygidial glands of dolichoderine and myrmicine ants have been considered to have alarm or defensive functions^31,52,53^. Our study experimentally confirms that Argentine ants actively utilize dolichodial and iridomyrmecin in the pygidial gland secretion for these functions, potentially facilitating a more rapid and coordinated group defense. Similar to the assays with dead harvester ants, Argentine ants increased their travel distance and time spent in the center zone when the glass bead in the center zone was treated with the iridoid(s). Also, Argentine ants increased their frequency of visitation to the center zone when the glass bead was treated with the iridoid(s).

Overall, the assays with fixed dead harvest ants and fixed glass beads showed similar results in most behavioral parameters (e.g., center zone distance, center zone cumulative duration, and center zone frequency). However, we found a distinct difference between these assays in the outcome with “latency to first (time until the first visit to the center zone)” parameter. Specifically, Argentine ants first visited the center zone more readily when the glass bead was treated with the pygidial gland chemicals, compared to the control. In contrast, there was no such effect of the pygidial gland chemicals on Argentine ant behavior when a dead harvester ant was used as the test item. Overall, Argentine ants responded quickly to the dead harvester ant fixed in the center, entering the center zone almost immediately after the assay initiation regardless of the presence of pygidial gland chemicals (e.g., 0.15 and 0.36 s until the first entry for PGE treatment and control, respectively). In contrast, when the clean glass bead was provided in the arena center, Argentine ants took 31.8 s on average to enter the center zone. This latency was substantially reduced (3.2–5.9 s) when the glass bead was treated with the pygidial gland chemical(s). These results suggest that some inherent differences between the test items (i.e., dead harvester ant vs. glass bead) might be responsible for the discrepancy. First, it is possible that some pre-existing chemicals on the harvester ant might have influenced the behavior of Argentine ant, causing them to enter the center zone immediately. For example, Argentine ants might be able to sense *n*-dodecane and other pre-existing cuticular hydrocarbons of the dead harvester ants from a distance (i.e., 4.8–5 cm) and respond to them accordingly. In contrast, the use of inert/clean glass bead as the test item would have eliminated any possible chemical cues. Secondly, it is possible that Argentine ant might have responded to the visual stimuli provided by the dead harvester ants, promptly entering the center zone. In this case, the lack of color of the glass bead might substantially delay the first entry to the center zone by the Argentine ant. Several studies on orientation behavior of ants, including the Argentine ant, suggest that visual and chemical mechanism interplay in mediating orientation behaviors^54–56^.

The use of the same chemical for two or more functions in different contexts is known in several insect systems, and this phenomenon is referred to as semiochemical parsimony^57^. In several ant species, the defensive chemicals often have various other functions in other behavioral contexts such as aggregation^58^, alarm^50,59^, and recruitment^57^. The current study reports that Argentine ants utilize their pygidial gland secretions containing dolichodial and iridomyrmecin as defensive allomones against heterospecific competitors, but the same secretions also cause nearby conspecifics to be alerted and quickly aggregate for defense. It is possible that Argentine ants also rely on these semiochemicals during intraspecific aggression^60^. Furthermore, dolichodial and iridomyrmecin are known to function in other behavioral contexts of the Argentine ant, such as necrophoresis and recruitment^19,44^. This functional versatility of dolichodial and iridomyrmecin appears to be related to the quantities produced/released by Argentine ants. For example, only nanogram quantities (per centimeter of trail) of dolichodial and iridomyrmecin were found on the Argentine ant recruitment trails^19^ while the quantities of iridoids on the cuticular surface of harvester ants in the current study (after being attacked by several Argentine ants) were in microgram quantities (per harvester ant). Studies suggest that the recruitment trail is continuously and uniformly reinforced over a period of time and the concentration of the trail pheromone may be dependent on the resource quality^19,54,61^. Being able to parsimoniously utilize these iridoids in a variety of behavioral/ecological contexts (e.g., colony maintenance, recruitment, defense, and alarm) may contribute to the Argentine ants’ ability to outcompete several other ant species^57^. Recent studies which have examined the chemical ecology of the tawny crazy ant (*Nylanderia fulva*) attributed its ability to displace native ants to the species’ superior ability to utilize its limited set of semiochemicals for multiple functions such as defense, detoxification, and recruitment to food resources or sites of conflict^62,63^. This suggests that semiochemical parsimony might play a significant role in the success of several invasive ant species worldwide.

## Materials and Methods

### Insects

Argentine ants were collected from the biological control citrus grove on the University of California, Riverside campus. Ant nests were excavated and transported to the laboratory, where the ants were extracted from the soil^64^. As the soil dried, workers moved the entire colony into moistened disks made of plaster of paris where they were carefully shaken off into plastic boxes (26.5 by 30 by 10 cm). Laboratory colonies were maintained in plastic boxes (26.5 by 30 by 10 cm); the inner sides were coated with Teflon® (Fluoropolymer emulsion, type 30, DuPont Polymers, Wilmington, DE) to prevent ants from escaping. Each colony was provided with two or three artificial nests constructed from plaster-filled petri dishes (9 cm in diameter by 1.5 cm in depth) with a smaller cylindrical area (5 cm in diameter by 1 cm depth) in the center of the dish, to serve as a nesting space. The colonies had free access to water and 25% (wt:vol) sucrose-water solution. Freshly killed American cockroaches (*Periplaneta americana*) were provided to the colonies three times a week as a protein source.

A nest of *P*. *californicus* was located on the University of California, Riverside campus. Foragers were collected from the soil surface around their trails or nest entrances. The collected harvester ants were temporarily kept in plastic boxes (26.5 by 30 by 10 cm) with the inner sides coated with Teflon®, before being used for the bioassays on the same day.

### Chemicals

The authentic standard of *trans*,*trans-*dolichodial (>99% pure) was collected from *Anisomorpha buprestoides* stick insect defensive secretion^65^ and subsequently cleaned. The authentic standard *cis*,*trans-*iridomyrmecin (94% pure) was synthesized from *cis*,*tran*-nepetalactone (see Supplementary Materials and Supplementary Fig. 1 for the details). Authentic standards of dolichodial, iridomyrmecin, and *n-*dodecane (Sigma-Aldrich) were used for identification of the volatile compounds and subsequent behavioral assays. The authentic standards of dolichodial (0.325 µg/ml) and iridomyrmecin (1 mg/ml) were originally obtained in methylene chloride and acetone, respectively. Thus, the same solvent was used to prepare the diluted samples for each of the standards to avoid any uncontrolled change or loss of the standard compounds during the evaporation of the original solvent and reconstitution in a new solvent. Also, a mixture of methylene chloride and acetone was used as the solvent control in some of the behavioral assays.

### Headspace volatile analyses

Headspace volatile analyses were conducted to determine if the pygidial gland chemicals are emitted by gaster-bending Argentine ants during aggressive interactions with a harvester ant. Samples (i.e., presence of aggressive interactions) were prepared and analyzed in the following manner. Five Argentine ants were briefly anesthetized with carbon dioxide and placed in a 2-ml glass vial (Agilent Technologies) coated with Teflon®. Once the ants recovered from the anesthesia (after 10 min), one live harvester ant was placed in the vial. The behavioral interactions between the two species were observed. Once any Argentine ant performed the gaster bending behavior on the harvester ant, a solid phase microextraction (SPME) fiber [Supelco Inc., 100-mm polydimethylsiloxane (PDMS)] was exposed in the headspace of the glass vial through the septum vial cap. Tip of the fiber was positioned at a consistent height - 15 mm from the vial bottom. Once gaster bending ceased (up to 2 min) the SPME fiber was removed from the vial and immediately injected into a coupled gas chromatograph-mass spectrometer (GC-MS) for analysis. For GC-MS, electron impact mass spectra (70 eV) were taken with an Agilent 5975 C mass selective detector interfaced to an Agilent 7890 A gas chromatograph fitted with a DB-5 column (30 m × 0.25 mm inner diameter, Agilent Technologies). Samples were injected in splitless mode, with a temperature program of 50 °C for 1 min, then 10 °C min^−1^ to 280 °C with a 5-min hold. Helium was used as the carrier gas. The compounds were identified based on comparisons (mass spectra and retention times) with standards of natural (dolichodial) or synthetic (iridomyrmecin) origin^19^. The analysis was replicated 10 times with the worker ants that were randomly selected from the colony boxes (for the Argentine ant, all of the ants used were active foragers collected outside of the nest). To confirm if detectable amounts of pygidial gland volatiles are produced only during aggressive interactions between two species, the headspace volatiles of control samples (i.e., absence of aggressive interactions) were also analyzed and compared with the treatment. These control samples were: 1) five Argentine ants only, 2) one harvester ant only, and 3) empty glass vial with no ants. Five replications were made for each of these controls and the collection time with the SPME fiber was 2 min for each sample. For Argentine ant only samples, the ants were anesthetized with carbon dioxide and a 10-min recovery period was allowed prior to the volatile collection. All ants were used only once for the headspace volatile analyses.

### Cuticular extract analyses

Cuticular extracts of the harvester ants were analyzed to determine if the pygidial gland chemicals were applied to them by the gaster-bending Argentine ants during aggressive interactions. A group of 100 Argentine ants was briefly anesthetized using carbon dioxide and placed in a glass petri dish (150 mm in diameter by 20 mm in height). A thin layer of Teflon® was applied to the inner side of the dish to prevent the ants from escaping. After the Argentine ants recovered from the anesthesia (approximately 10 min), one harvester ant was placed in the dish. From the time point when an Argentine ant showed the first gaster bending towards the harvester ant, the ants were allowed to continue interacting for an additional 2 min. During this period, multiple Argentine ants showed gaster bending responses. The total number of gaster bending responses was recorded. After the 2-min observation, the harvester ant was removed from the glass petri dish and immediately placed on dry ice. Dry ice killed the harvester ant instantly and kept it at a low temperature (−78.5 °C), potentially limiting dissipation of any volatile chemicals from its cuticle.

Thirty harvester ants were individually prepared as described above, and pooled for subsequent solvent extractions. New set of 100 Argentine ants was used for each harvester ant sample. The pooled ant sample was loaded on top of 150 mg of silica gel (60 Å pore size, 230–400 mesh, Whatman®) in a glass Pasteur pipette (7 mm in diameter by 14.6 cm in length, Fisher Scientific) for brief extraction and simultaneous fractionation of the extracted chemicals. Two fractions were obtained from the sample. Fraction 1 (non-polar fraction) was obtained by flushing the ants and the column with 2 ml of hexane; Fraction 2 (polar fraction) was obtained by subsequently flushing the column with 2 ml of methylene chloride. Each extraction/fractionation process was brief, allowing the solvent to contact the sample for about 5–10 s. The fractions were collected in separate vials, and concentrated to 0.5 ml under nitrogen gas. Both fractions were examined with normal phase (silica gel) thin layer chromatography (Flexible plates for TLC AL SIL G/UV 250 µm layer, Whatman®). Both fractions were further analyzed with an Agilent 7890 gas chromatograph equipped with a DB-5 column (30 m × 0.25 mm inner diameter, Agilent Technologies) and a flame ionization detector (GC-FID), using an automatic liquid sampler (ALS) that injected 1 µl of sample. Samples were injected in split mode, with a temperature program of 50 °C for 1 min and then 10 °C min^−1^ to 300 °C with a 10-min hold. Helium was used as the carrier gas. In addition to the GC-FID analysis, the samples were also analyzed with a GC-MS using the methods described in the headspace volatiles analysis section. The compounds were identified based on comparisons (mass spectra and retention times) with the standards^19^. A control sample was prepared from 30 harvester ants that had not interacted with Argentine ants, but were otherwise handled identically.

To estimate the quantities of the compounds per harvester ant, a series of external standards of known concentrations (0.01, 0.02, 0.04, 0.08, 0.16, and 0.32 µg/µl for dolichodial in methylene chloride; 0.0625, 0.125, 0.25, 0.5, and 1 µg/µl for iridomyrmecin in acetone) were analyzed with the GC-FID. Calibration curves were established for each compound series, and the total quantity of each compound in the cuticular extract was estimated based on the corresponding calibration curve. The estimated quantities of the chemicals in the cuticular extract were within the concentration ranges of standards used to generate the corresponding calibration curves. To obtain the quantities per harvester ant, the values estimated for the entire sample (30 harvester ants combined) were divided by 30, assuming that each of the harvester ants contributed equally to the total quantities.

### Collection and chemical analyses of pygidial gland chemicals

The pygidial gland secretion was collected from live Argentine ants for subsequent behavioral assays. First, a group of Argentine ants were briefly anesthetized with carbon dioxide and subsequently chilled on an ice pack. The pygidial gland secretion was “milked” out of the gland reservoir of each ant by gently squeezing the dorsal posterior region of the gaster with a pair of fine forceps. The droplet of pygidial gland secretion (referred to as “pygidial gland extract” or “PGE”) was taken up into a glass capillary micropipette (1 µl in capacity, Drummond Scientific Co.) and weighed. Ten separate collections were made from ten different Argentine ants as replicates. The ten collections of PGE were pooled by rinsing them out of the capillary with acetone (0.5 ml). The pooled sample was analyzed with an Agilent 7890 gas chromatograph equipped with a DB-5 column (30 m × 0.25 mm inner diameter, Agilent Technologies) and a flame ionization detector (GC-FID), using an automatic liquid sampler (ALS) that injected 1 µl of sample. The amounts of dolichodial and iridomyrmecin in one ant equivalent of PGE were estimated with the calibration curves established with a series of external standards of known concentrations.

### Response of harvester ants when topically treated with pygidial gland chemicals

The behavioral effect of Argentine ant pygidial gland chemicals to harvester ants was examined by topically applying PGE on the cuticular surface of the harvester ants. Using a pipette pump, one ant equivalent of PGE was dispensed from the glass micropipette and applied onto the head of the harvester ant, between the compound eyes. The head was chosen as the location for PGE treatment because Argentine ants typically aim their gasters towards the opponent’s head during gaster bending^27^. The control consisted of harvester ants that were not treated with PGE, but otherwise handled identically. The treatment or control harvester ant was immediately placed in a single well of a 24-well cell culture plate (Corning Inc.) and the behavioral responses of the harvester ant were recorded for 30 min using a video camera (Canon FS11, Canon Inc.). Mortality was recorded after 24 h. Treatment and control were replicated 10 times each.

The recorded video footages were used to analyze the behavioral responses of the harvester ants. Several distinctive behavioral categories were defined based on pilot laboratory observations (data not shown). The behavioral categories were: uninhibited mobility (UM), mandible clasping (C), mandible clasping with gaster manipulation (CG), grooming (G), grooming with gaster manipulation (GG), turning upside down (UP, insect rolls on its dorsal side), and knockout (KO, insect ceases locomotion coupled with leg twitches). The recorded video footage was repeatedly observed on a separate monitor. Using the time information associated with the recorded footage, total duration (s) for each behavioral category was recorded. For the data interpretation, we considered the categories C, CG, G, and GG as signs of irritation, and the categories UP and KO as signs of insecticidal activity.

### Response of Argentine ants to items treated with pygidial gland chemicals

The effects of pygidial gland chemicals on the behavior of Argentine ants were studied by analyzing their responses towards inanimate objects treated with the chemicals. In the first assay, a dead California harvester ant was used as a test item. The dead harvester ant was fixed in the center of the assay arena with an adhesive (Elmer’s School Glue®, Borden, Inc., Columbus Oh), preventing the movement of the test item (and the source of the chemicals) by the focal Argentine ant. With this method, several behavioral parameters that would be significantly influenced by the locational shift of the test items (e.g., travel distance or velocity in certain zones within the arena) could be collected and analyzed in a standardized manner. In the second assay, a dead California harvester ant was used again, but without being fixed in the center of the arena. By allowing the dead harvester ant to be moved from its original place, some of the consequences of the behavioral modifications of the Argentine ants (as they were seen in the first assay) could be observed and quantified. In the third assay, a clean glass bead fixed in the center of the arena was used as a test item. The use of a chemically inert, clear glass bead (3 mm in diameter) as a test item allowed us to examine the behavioral effects of the pygidial gland chemicals with a minimal influence of other preexisting stimuli such as visual, chemical, and tactile cues. With the glass bead as a test item, authentic standards of two iridoids (as individual compounds or a mixture) were also tested in addition to the crude pygidial gland secretion (PGE) obtained from the Argentine ants.

In all assays, the behavior of a single Argentine ant was observed in a circular arena (11 cm in diameter) with a plastic wall. The inner surface of the wall was coated with Teflon® to prevent the ant from escaping. The bottom of the arena was lined with a filter paper disc (15 cm in diameter, GE Healthcare Life Sciences, Whatman^TM^). The arena bottom included two zones - the center and outer zones. The center zone consisted of a central circular area (3.5 cm in diameter) and the outer zone consisted of the remaining radial area surrounding the center zone. For the assays with dead harvester ants as test items, a single freeze-killed harvester ant was placed in the center of the arena with or without the adhesive. For the assays with glass beads as test items, the beads were fixed in the center of the arena with the adhesive. Completed arenas were left for 24 h prior to an experiment to ensure the complete hardening of the adhesive.

Immediately before initiating the assays, the chemicals were applied on the test items. For the assays with dead harvester ants as test items, one ant equivalent of crude PGE (total weight ≈17 µg containing ≈0.05 µg of dolichodial and ≈0.15 µg of iridomyrmecin; see Results) was dispensed from the glass micropipette and applied onto the head of the harvester ant, between the compound eyes (PGE). Control consisted of dead harvester ants without PGE treatment. For the assays with glass beads as test items, the following treatments were tested: (1) application of one ant equivalent of PGE immediately followed by 1 µl of solvent (methylene chloride and acetone solvent mixture at 1:1, vol:vol) (PGE), (2) application of standard mixture containing 0.08 µg dolichodial and 0.25 µg iridomyrmecin in 1 µl solvent mixture (Mix), (3) application of 0.08 µg dolichodial only in 1 µl methylene chloride (Dol), and (4) application of 0.25 µg iridomyrmecin only in 1 µl acetone (Irid). The quantities of the standard iridoids were based on the quantities estimated for one harvester ant in the cuticular extract analyses, and they were also comparable with the quantities found in one ant equivalent of PGE (see Results). The test items treated with solvent only served as the control.

One Argentine ant was arbitrarily selected among the active foragers in the main laboratory colonies and subsequently confined at the release point within the assay arena (3 cm away from perimeter of the center zone) using a small plastic ring (1 cm in diameter) coated with Teflon®. After a 5-min acclimation period, the chemical treatment was made to the test items, and the Argentine ant was immediately released by lifting the small plastic ring confinement. Behavioral responses were recorded for 10 min per trial using EthoVision XT video tracking system (see Behavioral analyses). Argentine ants were used only once, and treatment and control were replicated 10 times each.

### Behavioral analyses

Because many different factors can affect the type and intensity of the alarm reaction observed in a biological system, a range of behaviors are generally provided to define an alarm behavior instead of relying on a specific definition^51^. In addition, behavioral responses can vary depending upon a variety of factors, including concentration of pheromone, compounds within the pheromone, colony size, and the spatial context of communication^3,66^. Given the complexity of defining the alarm reactions caused by the alarm pheromone, we used a computerized behavior tracking system in combination with manual observation, enabling quantitative analyses for some of the qualitative parameters.

A computerized behavior tracking system, EthoVision XT version 11.0.928 (Noldus Information Technology), was used to capture video images of each trial and record behavioral parameters of interest. The detection method used for acquisition was dynamic subtraction as described below. The detection thresholds for Argentine ant tracking were set so that all objects that were different from the background image by less than 40 or greater than 600 pixels were ignored and therefore recognized as part of the background. The sample rate (rate at which EthoVision analyzes the images to find the subject) was 25 samples s^−1^. Only the samples with <1% missed samples and <1% subject not found (no subject detected by the EthoVision) were selected. Velocity or speed (distance moved per unit time) was calculated as cm s^−1^.

For all three behavioral assays with Argentine ants, overall speed (total velocity in entire arena, cm s^−1^) and overall distance (total distance traveled in entire arena, cm) were recorded to determine the overall activity levels of the focal Argentine ants within the experimental arena. For the assays with test items (dead harvester ant or glass bead) fixed in the center of the arena, the following behavioral parameters were recorded: center zone distance (cm), center zone velocity (cm s^−1^), center zone cumulative duration (total time spent in center zone, s), center zone frequency (total number of visits to center zone), and latency to first (time until the first visit to the center zone, s). All parameters were automatically recorded with EthoVision XT software. For the assays with a dead harvester ant unfixed in the arena center, the following parameters were manually recorded based on the recorded video footage: total number of physical contacts between the Argentine ant and the dead harvester ant while Argentine ant performing antennation, mandible clasping, gaster bending, or crawling responses, total duration of the physical contacts, and total number of times the Argentine ant moved the dead harvester ant.

### Statistical analyses

To compare relative quantities of dolichodial and iridomyrmecin between different samples in the headspace volatile analyses, the areas of the corresponding peaks in the chromatograms were compared^67^. A Wilcoxon rank sum test was used due to small (n < 10) and unequal sample sizes^68^.

To analyze the differences in total time spent in the behavioral categories between treatment and control groups of harvester ants (i.e., with or without topical treatment with PGE), a permutational MANOVA (multivariate analysis of variance) was performed with the Adonis function in the R package Vegan^69^. The permutational MANOVA is analogous to traditional MANOVA, but more robust to violations of normality assumptions^70^. Principal component analysis (PCA) was also performed using the R package FactoMineR^71^ to identify behavioral categories that are correlated with the treatment group.

For the Argentine ant behavioral assays with dead harvester ants, depending on normality and homogeneity of variance of the data, a two-sided two-sample t-test, two-sided Welch two-sample t-test, or a two-sided Wilcoxon rank sum test was performed to compare PGE treatments and controls for each of the behavioral parameters. Due to their non-normal distribution, the data from the Argentine ant behavioral assays with glass beads were analyzed using a Kruskal-Wallis test, followed by Dunn’s test for all-pairwise comparisons of mean ranks with a Bonferroni adjustment at α = 0.001 using the “PMCMR” package. All statistical tests were performed on R Statistical software^72^.

### Data Availability

All data generated or analyzed during this study are included in this published article (and its Supplementary Materials).

## Electronic supplementary material

Supplementary materials
