# Supplementary material for: Verification of Argentine ant defensive compounds and their behavioral effects on heterospecific competitors and conspecific nestmates
Source: Sci Rep. 2018 Jan 24;8:1477. doi: 10.1038/s41598-018-19435-6 (PMC5784131; doi:10.1038/s41598-018-19435-6)
Supplement: Supplementary file 1 — Supplementary materials [file 41598_2018_19435_MOESM1_ESM.pdf]

Verification of Argentine ant defensive compounds and their behavioral effects on heterospecific competitors and conspecific nestmates

Kevin F. Welzel[1], Shao Hung Lee[2], Aaron T. Dossey[3], Kamlesh R. Chauhan[4], and Dong-Hwan Choe[5]

[1]Department of Entomology, University of California, Riverside, Riverside, CA 92521, USA.
[2]Department of Entomology, University of California, Riverside, Riverside, CA 92521, USA.
[3]All Things Bugs LLC. 2211 Windsong Dr., Midwest City, OK 73130, USA.
[4]Invasive Insects Biocontrol and Behavior Laboratory, USDA- ARS, BARC- West Bldg. 007, 10300 Baltimore Avenue, Beltsville, MD 20705, USA.
[5]Department of Entomology, University of California, Riverside, Riverside, CA 92521, USA.

Correspondence and request for materials should be addressed to K.F.W. (email: kwelz001@ucr.edu)

Supplementary Materials

Methods

Chemicals. The authentic standard of *trans,trans*-dolichodial (**1** in Supplementary Fig. 1) [referred to as ‘‘peruphasmal’’ in Dossey et al. (2006)] (>99% pure) was collected from a "peruphasmal-producing" field population of *Anisomorpha buprestoides* stick insect (Dossey et al. 2008, Dossey 2010). At least 20 adult females were milked to obtain 100-150 µl of crude defensive secretions in a single 4 mL borosilicate glass vial with a Teflon® lined screw cap. Initial chemical verification of *trans,trans*-dolichodial was conducted via 1D NMR. The crude sample was extracted 3 times via vigorous mechanized vortexing with approximately 1 mL of methylene chloride (HPLC Grade) each time. The 3 extracts were pooled and residual moisture was removed with anhydrous magnesium sulfate. The sample was then filtered to remove the magnesium sulfate/water complex, and placed into a tared 4 mL wide mouthed borosilicate glass vial with a Teflon lined screw cap. The solvent was then removed under a gentle stream of clean nitrogen gas (≈1 h), leaving 9.9 mg of "neat" pure *trans,trans*-dolichodial (by diastereomer, absolute stereochemistry or purity was not verified) in the vial. The chemical identity was verified by 1D NMR and GC-MS. The sample was dissolved in 0.5 ml of methylene chloride and stored in a closed vial at -20 °C until used.

Synthetic *cis,trans*-iridomyrmecin (94% pure) was prepared by following reported procedure (Chauhan and Schmidt 2014). Availability of *cis,trans*-nepetalactone (**3** in Supplementary Fig. 1) (4aS,7S,7aR), a major component of catnip oil (*Nepeta cataria*), was the key to this synthetic approach. Methanolysis of *cis,trans*-nepetalactone at room temperature in 5% methanolic $NaHCO_3$ solution (95:5 methanol/water in volume) gave an isomeric mixture

of methyl ester-aldehyde (**4** in Supplementary Fig. 1), which was quantitatively protected to the cyclic acetals by azeotropic dehydration with ethane-1,2-diol and then followed by diisobutylaluminium hydride (DIBAL) reduction converting ester to hydroxyl moiety (**5** in Supplementary Fig. 1) to 94% yield over the two steps. Desired enantiomer of the cyclic acetal was separated by flash column chromatography. Deprotection of the cyclic acetal was carried out under mild acidic hydrolysis at room temperature to quantitative yields of free hydroxyl aldehyde (**6** in Supplementary Fig. 1), which was oxidized to conclude the synthesis of iridomyrmecin (**2** in Supplementary Fig. 1) in 62%. The purity and identity of the compound was checked with 1D NMR and GC-MS.

Figures

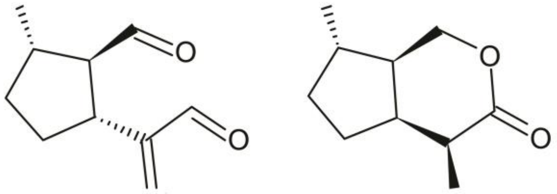

1: Dolichodial **2:** Iridomyrmecin

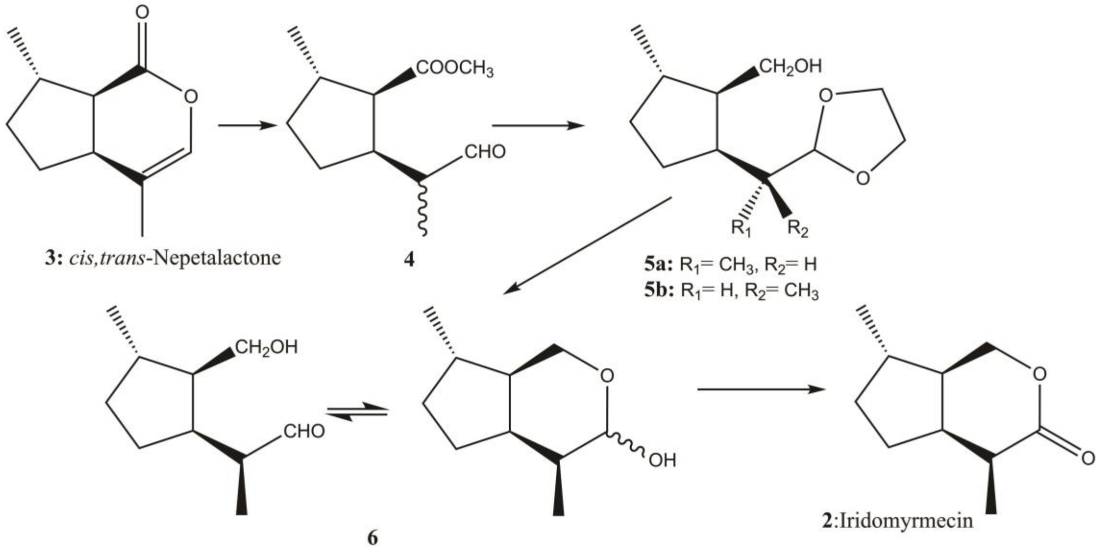

Supplementary Figure 1. Authentic standards of *trans,trans*-dolichodial and *cis,trans*-iridomyrmecin. Chemical structures of dolichodial (**1**) and iridomyrmecin (**2**), and synthesis procedure for iridomyrmecin are shown.

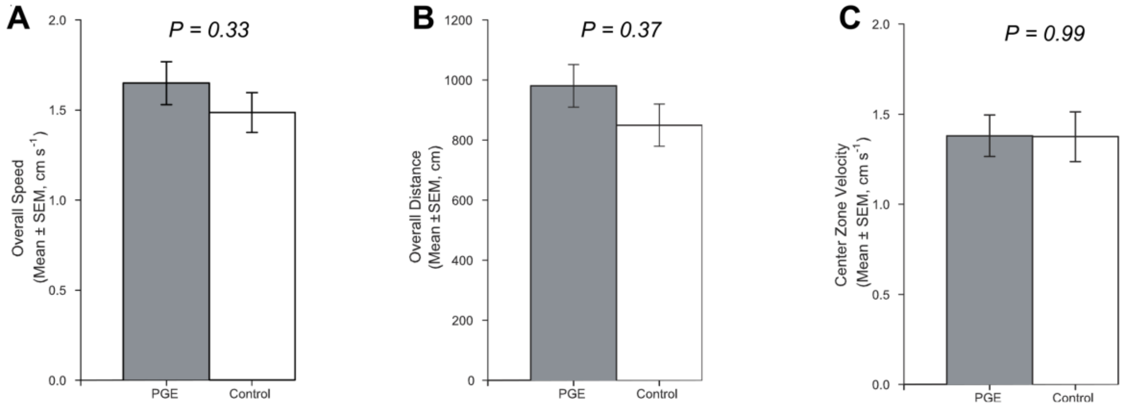

Supplementary Figure 2. Responses of an Argentine ant to a fixed dead harvester ant with or without Argentine ant pygidial gland extract (PGE). Ten replications were made for PGE treatment (grey) and control (white). The behavioral parameters analyzed include: overall speed (A), overall travel distance (B), and center zone velocity (C). P-value is provided for each behavioral parameter (see results for the statistical methods used). Error bars indicate SEM values.

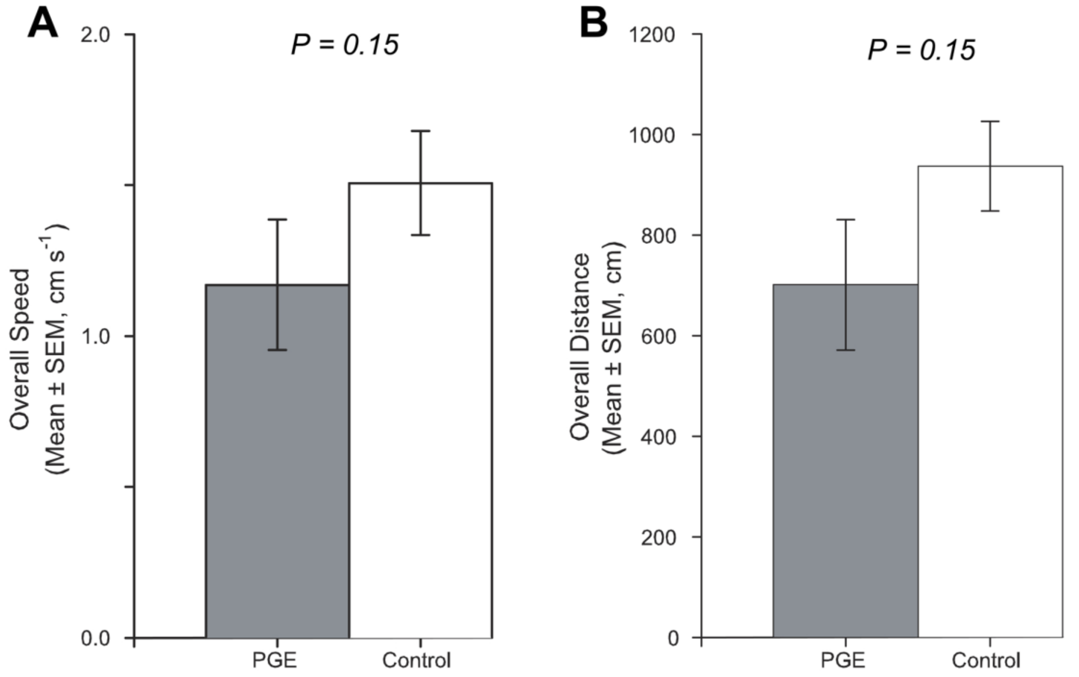

Supplementary Figure 3. Response of an Argentine ant to an unfixed dead harvester ant with or without Argentine ant pygidial gland extract (PGE). Ten replications were made for PGE treatment (grey) and solvent only control (white). The Behavioral parameters analyzed include: number of overall speed (A), and overall travel distance (B). P-value is provided for each behavioral parameter (see results for the statistical methods used). Error bars indicate SEM values.

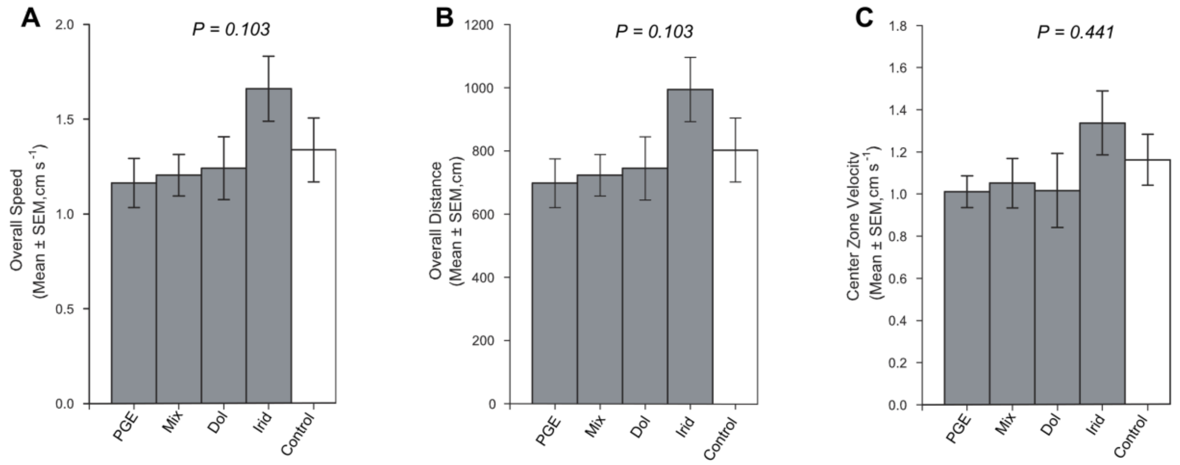

Supplementary Figure 4. Response of an Argentine ant to a glass bead treated with pygidial gland extract (PGE), a mixture of authentic standards of dolichodial and iridomyrmecin (Mix), dolichodial standard (Dol), iridomyrmecin standard (Irid), and solvent only (Control). Ten replications were made for each of the treatments (grey) and control (white). The behavioral parameters analyzed include: overall speed (A), overall travel distance (B), and center zone velocity (C). For statistical analysis see results. P-value is provided for each behavioral parameter (see results for the statistical methods used). Error bars indicate SEM values.

Table

Supplementary Table 1. Means (± SEM) associated with five treatment groups for the seven Argentine ant's behavioral response parameters in assays with a fixed glass bead. Treatments include pygidial gland extract (PGE), a mixture of authentic standards of dolichodial and iridomyrmecin (Mix), dolichodial standard (Dol), and iridomyrmecin standard (Irid). The control consisted of solvent only (Control). Treatment means followed by the letter "a" are significantly different from "b" by all-pairwise comparisons of mean ranks with a Bonferroni correction at α = 0.001 (see results for the statistical methods used).

Treatment	Overall Speed ($cm\ s^{-1}$)	Overall Distance (cm)	Center Zone Distance (cm)	Center Zone Velocity ($cm\ s^{-1}$)	Center Zone Cumulative Duration (s)	Center Zone Frequency	Latency to First (s)
**PGE**	1.16 ± 0.17	697.6 ± 77.4	99.4 ± 6.5 a	1.01 ± 0.08	101.2 ± 2.1 a	28.7 ± 2.1 a	5.90 ± 1.25 a
**Mix**	1.20 ± 0.13	722.5 ± 65.4	117.0 ± 10.3 a	1.05 ± 0.12	126.7 ± 15.5 a	29.3 ± 2.1 a	3.22 ± 0.89 a
**Dol**	1.24 ± 0.17	744.4 ± 99.5	98.0 ± 7.6 a	1.02 ± 0.18	137.6 ± 38.1 a	29.1 ± 3.7 a	5.73 ± 2.56 a
**Irid**	1.66 ± 0.17	1007.2 ± 101.9	134.1 ± 15.3 a	1.34 ± 0.15	108.5 ± 15.6 a	33.6 ± 3.6 a	5.12 ± 2.33 a
**Control**	1.38 ± 0.17	802.3 ± 101.0	55.7 ± 6.1 b	1.16 ± 0.12	59.5 ± 6.5 b	16.2 ± 2.0 b	31.8 ± 6.1 b

References Cited

Chauhan, K. R. & Schmidt, W. Biorational synthesis of iridomyrmecin diastereomers from catnip oil. *Tetrahedron Lett*. **55**, 2534-2536 (2014)

Dossey A. T., Walse S. S., Rocca J. R. & Edison A. S. Single insect NMR: A new tool to probe chemical biodiversity. *ACS Chem. Biol.* **1**, 511–514 (2006)

Dossey, A. T., Walse, S. S. & Edison, A. S. Developmental and geographical variation in the chemical defense of the walkingstick insect *Anisomorpha buprestoides*. *J. Chem. Ecol.* **34**, 584-590 (2008)

Dossey, A. T. Insects and their chemical weaponry: new potential for drug discovery. *Nat. Prod. Rep.* **27**, 1737-1757 (2010)
